# An IoT-Enabled mHealth Sensing Approach for Remote Detection of Keratoconus Using Smartphone Technology

**DOI:** 10.3390/s25051316

**Published:** 2025-02-21

**Authors:** Behnam Askarian, Amin Askarian, Fatemehsadat Tabei, Jo Woon Chong

**Affiliations:** 1 College of Engineering, West Texas A&M University, Canyon, TX 79016, USA; ftabei@wtamu.edu; 2 Askarian Clinic, Shiraz 71877-75778, Iran; askarian_sums@yahoo.com; 3 Department of Electrical and Computer Engineering, Texas Tech University, Lubbock, TX 79409, USA; j.chong@ttu.edu

**Keywords:** keratoconus (KC), corneal topography, smartphone, Placido disc, support vector machine (SVM)

## Abstract

Keratoconus (KC) is a progressive eye disease and a major cause of vision impairment and blindness worldwide. Early diagnosis is crucial for effective management, yet conventional diagnostic methods rely on expensive and bulky imaging devices, limiting accessibility, especially in resource-constrained settings. This paper introduces a novel smartphone-based approach for the early detection of KC, leveraging screen-projected Placido disc patterns and an advanced image processing framework. Unlike traditional corneal topographers, our method utilizes a unique Placido disc projection technique and a machine learning-based classification model to analyze corneal irregularities with high precision. With a sensitivity of 96.08%, specificity of 97.96%, and overall accuracy of 97% on our dataset, the proposed system demonstrates exceptional diagnostic reliability. By transforming a standard smartphone into an effective screening tool, this innovation provides an affordable, portable, and user-friendly solution for early KC detection, bridging the gap in eye care accessibility and reducing the global burden of undiagnosed keratoconus.

## 1. Introduction

**Figure 1:**
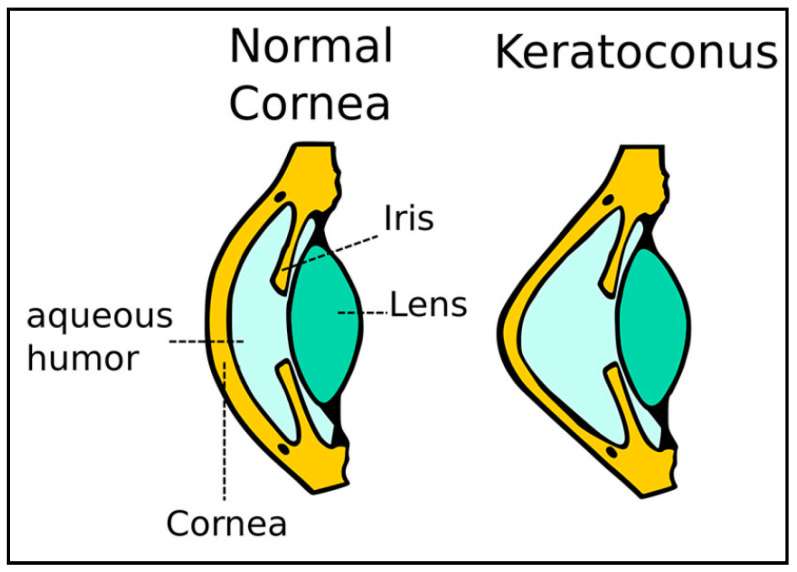
Demonstration of healthy and KC eye: left, healthy cornea, and right, KC eye with cone-shaped cornea [6].

Keratoconus (KC) is a corneal disorder characterized by the progressive thinning and steepening of the cornea, resulting in blurred and distorted vision (refer to Figure 1) [1,2]. The etiological factors associated with keratoconus (KC) include Down syndrome, atopy, eczema, excessive eye rubbing, and various genetic disorders [3]. Furthermore, environmental factors, including prolonged exposure to ultraviolet light and elevated temperatures prevalent in certain Asian countries such as India, contribute to a higher incidence of keratoconus (KC) subjects when compared to other regions of the world [4,5].

The primary method for diagnosing keratoconus (KC) is through monitoring clinical signs, including Vogt striae, the Rizzuti sign, the Munson sign, Fleischer rings, and the retinoscopy scissoring reflex, all observed during a slit-lamp examination [7]. These methodologies are effective for the diagnosis of moderate to advanced cases of keratoconus (KC). However, for the identification of mild stages of KC and to achieve a more precise diagnosis, it is essential to conduct a comprehensive examination of corneal features, which includes assessments of topography, elevation, thickness, and biomechanical characteristics [8,9]. Utilizing corneal topography along with the Scheimpflug method is essential for this purpose.

Numerous studies have been conducted to detect keratoconus (KC) utilizing corneal topography data. These studies have demonstrated that significant insights regarding eyes affected by KC can be derived from both anterior features and geometrical lateral characteristics [10,11,12]. Most methodologies, however, depend on subjective interpretations of topographic maps, which may be influenced by the perspectives of the observers [13]. The application of machine learning analysis has been emphasized as one of the objective methodologies for the detection of Knowledge Components (KCs) [14,15,16]. Smolek and Klyce proposed a system for the identification of keratoconus (KC) utilizing corneal topography indices within a neural network framework [14]. Chastang et al. proposed a binary decision tree method using corneal topography indices to distinguish keratoconus (KC) eyes from healthy eyes [15]. The methods discussed utilized anterior topography characteristics to identify keratoconus (KC).

Additionally, advancements in technology have made pachymetric and corneal posterior curvature data available, which are used to evaluate corneal features and detect keratoconus [17]. Perez et al. conducted a study in which they combined data from various corneal instruments, including Pentacam, Orbscan, and videokeratography. Their findings suggest that these methods can detect keratoconus (KC) in its early stages. However, they also noted that the false positive rate was elevated in their study. [18]. Fatemeh et al. [19] proposed an automatic KC detection method by adopting artificial intelligence. Their algorithm utilizes corneal topography images obtained using a Pentacam [20] that have been labeled by ophthalmologists as KC eyes and healthy eyes. The downside to their approach, though, is that a limited number of images were included in the training dataset.

One of the first studies to adopt neural networks for diagnosing KC was conducted by Perissutti et al. [21]. They reported a 92% accuracy level using neural network classification. Their research compares the integrated keratoconus (KC) detection approach with monocular and binocular methods. Arbelaez et al. proposed a classification algorithm for diagnosing keratoconus with high accuracy. In their classification model, the authors utilized corneal thickness and features of the posterior corneal surface to detect keratoconus (KC). The incorporation of these features significantly enhanced the algorithm’s sensitivity for subclinical diagnoses of KC. As a result, the classification algorithm is particularly valuable in identifying early symptoms associated with this condition. The reliability of the classifier developed by the authors was approximately 97.2%.

Early diagnosis of KC is crucial for effective disease treatment. Maeda et al. combined discriminatory analysis and classification trees to evaluate corneal topography data for diagnosing KC [13]. A sensitivity of 89% was achieved, which is lower than the 95% sensitivity attained by the Support Vector Machine (SVM) algorithm. In another study, researchers proposed mapping the anterior corneal surface using a seventh-order Zernike polynomial and incorporating a decision tree to differentiate between healthy eyes and those affected by keratoconus (KC) [16]. The method demonstrated sensitivity, precision, and accuracy rates of 92%, 93%, and 94%, respectively. Chastang et al. incorporated a decision tree classifier utilizing data from a corneal topography device, achieving an accuracy of 95% [15]. The drawback of their solution was the limited variety of input data.

All the aforementioned methods necessitate the use of advanced clinical diagnostic devices for data acquisition. However, these devices tend to be bulky, heavy, and costly, as well as requiring a skilled technician for proper maintenance and operation. Furthermore, ophthalmologists and optometrists should take into account other clinical signs of keratoconus simultaneously to ensure an accurate and reliable diagnosis of the condition [22,23]. Recent advancements in machine learning and image processing, along with the proliferation of smartphone technology, have led to the development of accurate and user-friendly imaging and diagnostic methods. As a result, smartphone applications have emerged as a promising new niche in healthcare for the screening and examination of ocular conditions. Our prior methodologies for smartphone-based diagnostics aimed at detecting ocular diseases have been articulated in [24,25,26,27] for detecting KC and cataracts.

While several keratoconus detection methods exist, they rely on sophisticated clinical instruments such as Scheimpflug imaging and corneal tomography, which provide high-resolution corneal mapping and precise curvature measurements [17,20]. These methods, though effective, require specialized training for operation and are costly, with devices such as the Pentacam HR exceeding USD 50,000, making them inaccessible in many regions, particularly in low-resource settings [28]. Traditional keratometry, while more affordable, lacks the ability to capture detailed posterior corneal curvature, which is critical for early keratoconus detection [13].

Existing smartphone-based approaches have attempted to bridge this gap by leveraging external attachments, such as slit-lamp adapters or external Placido disc mounts, to acquire corneal images [29,30]. However, these approaches introduce additional costs and complexity, limiting their adoption in non-clinical settings. Furthermore, many of these methods lack the accuracy of traditional imaging techniques due to inconsistencies in lighting, alignment, and device calibration [30].

This study fills this gap by proposing a fully standalone smartphone-based method that leverages Placido disc projections without additional hardware, offering a portable and cost-effective alternative. By integrating adaptive contrast mechanisms and machine learning-based classification, our approach enhances detection accuracy while ensuring accessibility for widespread deployment, particularly in underserved regions.

In this paper, we present a novel method for detecting keratoconus (KC) using a standalone smartphone-based approach that requires no external attachments or additional hardware. Our method utilizes the smartphone screen to generate Placido discs, which are directly projected onto the cornea while images are captured using the front camera. This approach eliminates the need for calibration or user training, enhancing accessibility and ease of use. The Placido rings automatically adjust based on screen size and eye distance, with customizable parameters such as number, radius, thickness, and color, ensuring adaptability for varying lighting conditions and environments. Furthermore, an adaptive contrast mechanism optimizes image quality for both indoor and outdoor settings.

To enhance robustness, we leverage the smartphone’s front-facing depth camera to dynamically adjust the size and angle of the Placido rings according to the user’s position. Image processing techniques are employed to extract corneal images and analyze the projected Placido discs. A Support Vector Machine (SVM) classifier is then used to distinguish between keratoconic and healthy eyes. Our luminance-based approach effectively mitigates variations in smartphone camera sensors and chromatic differences, making it independent of sensor color characteristics and eye pigmentation. To address data limitations, a 10-fold cross-validation procedure was implemented to rigorously evaluate the machine learning model. As a result, the proposed method achieved an impressive classification accuracy of 97%, demonstrating strong clinical viability.

The key contributions of this study are as follows:

- Standalone Smartphone-Based Detection: developed an innovative keratoconus detection method that requires no external attachments or specialized equipment.
- Placido Disc Projection via Smartphone Display: designed a novel method for generating and projecting Placido rings directly from the smartphone screen.
- Adaptive Contrast Enhancement: implemented an adaptive contrast mechanism to optimize image quality under diverse lighting conditions.
- Machine Learning-Based Classification: utilized SVM for accurate and automated classification of keratoconus.
- High Diagnostic Accuracy: achieved 97% accuracy, highlighting the potential for real-world clinical applications.

The remainder of this paper is organized as follows: Section 2 describes the materials and methods used in this study, including data acquisition, preprocessing techniques, Placido disc extraction, and classification methods. Section 3 presents the classification results and key image features associated with keratoconus detection. Section 4 discusses the implications of the findings, along with study limitations and future directions. Finally, Section 5 concludes this paper by summarizing the key contributions and potential impact of this research.

## 2. Materials and Methods

For data acquisition, we utilized an iPhone X smartphone. The iPhone X features a 7-megapixel CCD front camera sensor [30]. To evaluate the feasibility of the proposed method across different smartphone models, we first tested it on the iPhone X, a widely used device with a lower-quality camera compared to modern smartphones. This allowed us to assess whether the method could still produce reliable results under less optimal imaging conditions. Subsequently, we extended our testing to newer smartphone models, including the iPhone 13 Pro and iPhone 16 Pro, both of which demonstrated improved image quality and enhanced classification accuracy. The findings confirm that our approach is robust across different camera specifications, ensuring accessibility and scalability for a wide range of users.

To replicate KC, we employed a corneal disease eye model. The eye model utilized in this study was a GPI Anatomical Cornea Eye #2780 (5× Life Size) model [31]. The eye model included five corneas, representing four disease stages and one healthy cornea [32]. Moreover, we developed an image featuring nine circular Placido discs intended for display on a smartphone screen. This design effectively replicates the Placido discs utilized in keratoscopes. To minimize the impact of external lighting conditions, all images were captured in a controlled indoor environment with diffused lighting, avoiding direct reflections on the corneal surface. The Placido disc projection was designed with an adaptive contrast feature, ensuring clarity under varying light conditions. Future versions of this method may incorporate automatic exposure adjustment to further improve robustness in different environments. For consistent image acquisition, the smartphone was positioned approximately 20 cm from the eye, with the camera lens aligned perpendicularly to the corneal surface. Users ensured that the Placido disc projection was centered on the pupil and maintained a steady hand (or used a phone stand) to minimize motion artifacts. The front-facing camera, equipped with facial tracking, helped maintain alignment automatically.

**Figure 2:**
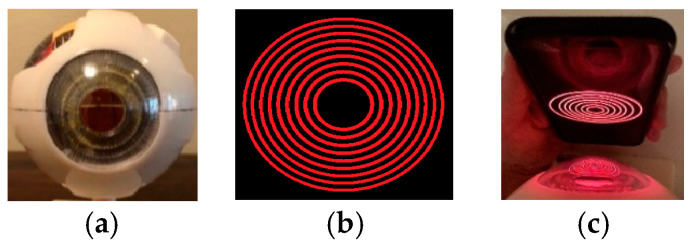
Materials used for data acquisition: (**a**) Axis model eye, (**b**) specially designed Placido discs (Red circles), and (**c**) projected Placido disc on the model eye using an iPhone X.

**Figure 3:**
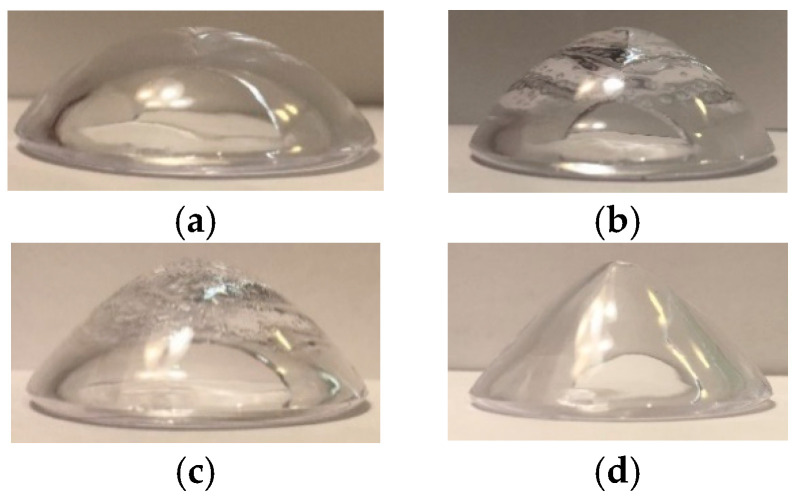
Demonstration of the four stages of keratoconus (KC) using the Axis eye model: (**a**) Stage 1 (mild) KC, (**b**) Stage 2 (moderate) KC, (**c**) Stage 3 (advanced) KC, and (**d**) Stage 4 (severe) KC.

The eye model utilized for data acquisition, along with the Placido disc, is illustrated in Figure 2. To simulate various stages of keratoconus (KC), we employed the KC emulated eye model, which features five corneas. These corneas represent four stages of KC, in addition to one healthy cornea. Figure 3 illustrates the four corneas utilized in the eye model. Specifically, Figure 3a depicts stage 1 (mild) keratoconus (KC), Figure 3b represents stage 2 (moderate) KC, Figure 3c showcases stage 3 (advanced) KC, and Figure 3d displays stage 4 (severe) KC. In total, 100 images were captured from the eye models, consisting of 50 images from healthy eyes and 50 images from diseased eyes.

**Figure 4:**
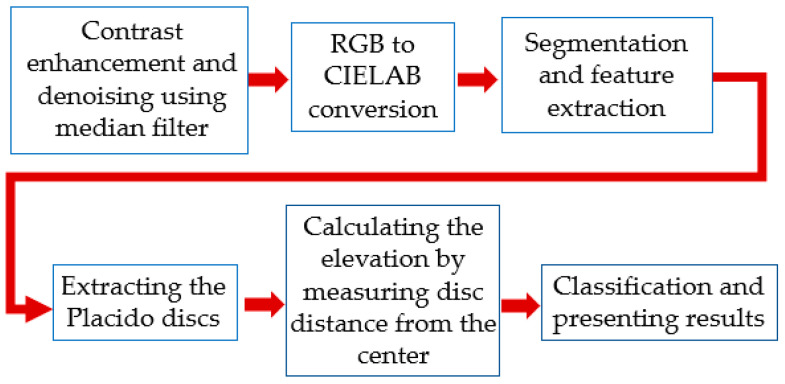
Flowchart of the proposed method.

Our proposed method begins with a preprocessing step, where the images are analyzed to reduce noise and enhance color contrast. Following this, the images are converted to the CIE LAB color space. Next, segmentation is performed to extract the regions of interest, specifically the Placido discs. The radii of the Placido discs are then measured, and the corresponding diopters are calculated. Finally, an SVM classifier is employed to differentiate between healthy eyes and those with keratoconus, and the results are presented. The steps of our proposed method are illustrated in Figure 4.

### 2.1. Preprocessing

**Figure 5:**
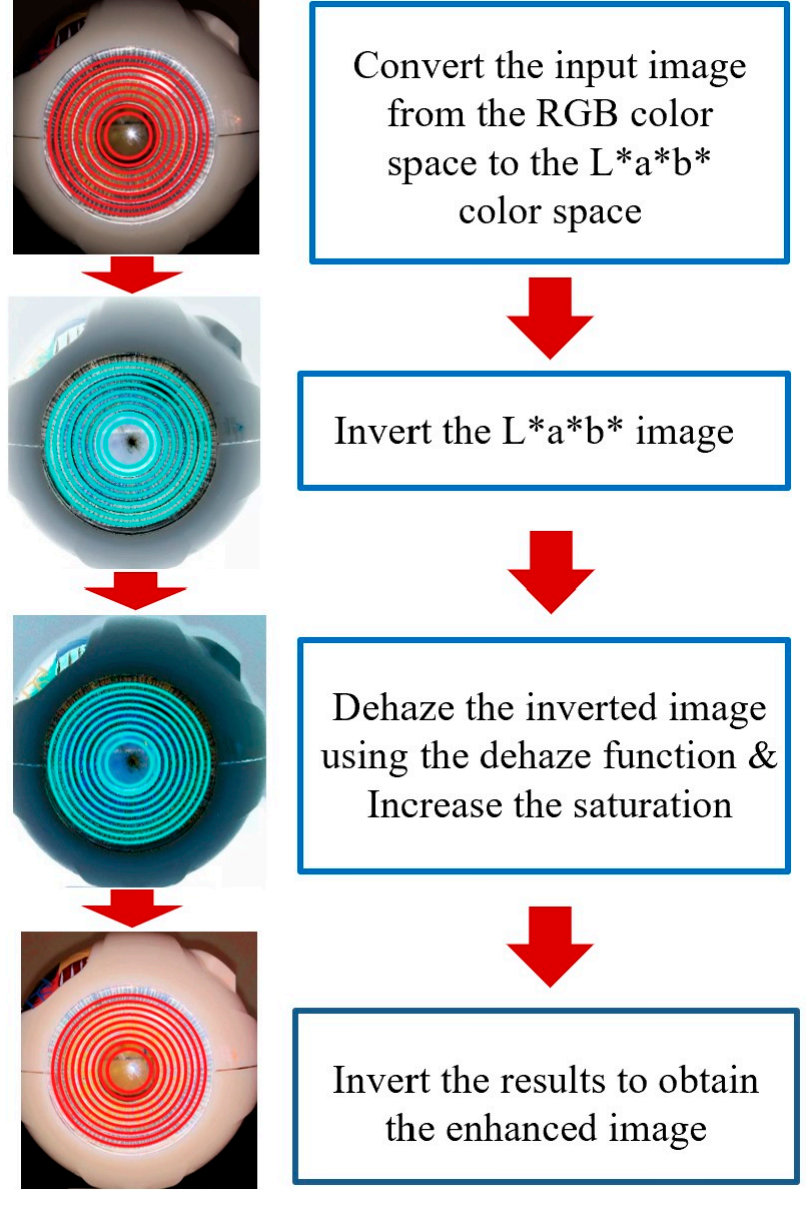
Visual representation of the color enhancement preprocessing stage, with corresponding images at each phase. The arrows illustrate the transitions between steps: (1) Convert RGB to Lab color space for better color separation, (2) Invert the Lab image to enhance features, (3) Apply dehazing and increase saturation for improved clarity and contrast, and (4) Invert back to restore natural colors with enhancements.

The preprocessing component in our proposed method is designed to denoise the data and enhance overall accuracy. This preprocessing phase consists of two primary steps: image denoising and contrast enhancement. The initial step involves applying a median filter to effectively denoise the image. Once the image is denoised, we proceed with color contrast enhancement to improve its quality. A flowchart illustrating the color enhancement procedure can be found in Figure 5. The central aspect of color contrast enhancement is image dehazing, which begins with modeling the image using the following equation:

*I*(*x*) = *J*(*x*) × *r*(*x*) + *A* (1 − *r* (*x*)), (1)

where *x* is a spatial location in the image, *I*(*x*) is the observed intensity at *x*, *A* is the environmental light, and *J* is the scene radiance. *r*(*x*) is the pixel intensity from the camera sensor at *x*. *J*(*x*) is object or scene intensity at *x*. *r*(*x*) indicates the emitted light percentage reflected (received) from the object or scene on the camera sensor [33,34]. The most important step of the haze removal algorithm is to estimate *A* and *r*(*x*) from the recorded image intensity *I*(*x*) so that *J*(*x*) can be recovered from *I*(*x*).

As previously noted, the primary objective of image haze removal is to recover J in Equation (1), which is expressed in the following equation:

(2) $$J\left(x\right)=\frac{I\left(x\right)-A}{\max\left(r\left(x\right),r_{0}\right)}+A$$

This model (Equation (2)) explains the haze effect and how it impacts the contrasts when the average image with a color contrast of A is extracted. By calculating the gradient magnitude of scene J through a uniform medium (*t*(*x*) = *t* < 1), the image contrast can be calculated using the following equation:

(3) $$\left\|\nabla I\right\|=\left\|t\nabla J\left(x\right)+\left(1-t\right)\nabla A\right\|$$

In order to minimize color distortion, conversion to the CIE LAB color space was employed. Initially, the input image in the RGB color space was transformed into the CIE LAB color space. Subsequently, the CIE LAB image underwent inversion, dehazing, and an increase in saturation. Finally, the image was inverted back to obtain the enhanced version. The flowchart illustrating this preprocessing step can be found in Figure 5, which depicts the image enhancement process applied to a sample emulated eye with Placido discs in a low-light environment.

### 2.2. Processing and Feature Extraction

#### 2.2.1. CIE LAB to Binary Conversion and Placido Disc Extraction

In the analysis of a cornea image, segmentation is initially employed to identify all foreground cornea pixels and extract the Placido discs. To delineate the Placido disc area within the corneal image, we implemented an innovative image processing technique that differentiates the designated areas based on their color features. We utilized the RGB color space for this purpose, allowing for effective identification of the specific regions.

**Figure 6:**
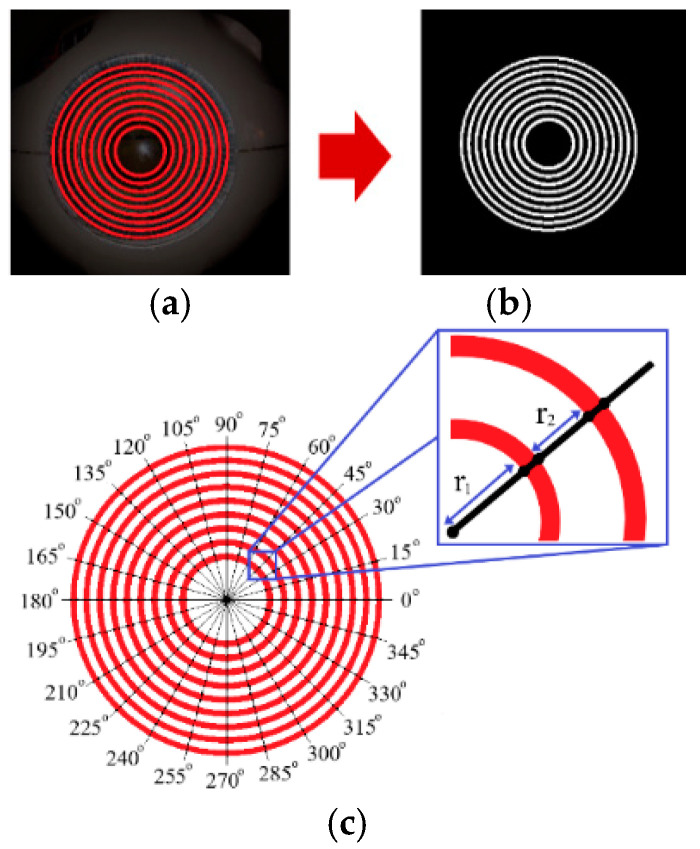
The outcomes of extracting the Placido disc from the corneal image using a CIE LAB color mask and radius feature extraction are as follows: (**a**) the Placido disc projected onto the corneal surface (red circles), (**b**) the extraction of the Placido discs from the corneal image, which have been converted into a binary format, and (**c**) the segmentation of the Placido discs (red circles) into 24 segments, covering angles from 0° to 360°, with each segment measuring 15°. In this picture, r represents the radial distance from the center, with r_1_ and r_2_ highlighting specific points along a radial line.

Once each pixel is located, its corresponding CIE LAB value is extracted. Table 1 presents the mean and standard deviation values for the Placido discs. Additionally, Figure 6 illustrates the results of the Placido disc extraction utilizing the color values provided in Table 1.

#### 2.2.2. Calculating Diopter from Placido Disc Radius

In corneal topography using Placido discs, the anterior surface of the cornea is assumed to behave as a convex mirror that reflects the concentric rings projected by the device. The pattern of these reflections provides insight into the corneal curvature, where deviations in ring spacing indicate variations in corneal shape. A steeper cornea causes the reflected rings to appear closer together, whereas a flatter cornea results in wider ring spacing. By analyzing these patterns, the Placido disc projection determines the radius of curvature (r) at different points on the corneal surface.

The radius of curvature is calculated using the mirror equation, and the corneal power, expressed in diopters (D), is determined using the keratometric equation [35]:

(4) $$D=\frac{n-1}{r}$$

where *r* is the radius of the Placido discs and *n* = 1.3375.

Since the diopter value is inversely proportional to the radius of curvature, a smaller *r* (steeper cornea) results in a higher diopter value, whereas a larger *r* (flatter cornea) corresponds to a lower diopter value.

This method allows for the identification of various corneal abnormalities. For example, regular astigmatism is characterized by two principal meridians with different curvature values, leading to a symmetrical toric shape. Irregular astigmatism, often caused by corneal scarring or diseases, results in a non-uniform curvature that distorts the topographic pattern. Keratoconus, a progressive corneal disorder, is detected by a localized steepening of the cornea, appearing as a high-diopter region on the topography map, often in the inferior or central portion of the cornea. The calculated diopter values are represented in color-coded topography maps, where different colors indicate varying curvature levels. Warmer colors (red and yellow) represent steeper corneal regions with higher diopter values, while cooler colors (green and blue) indicate flatter regions with lower diopter values. These maps provide a clear visualization of corneal shape and irregularities, aiding in the diagnosis, monitoring, and management of corneal conditions.

Overall, the diopter calculation in keratoscopic corneal topography plays a crucial role in understanding corneal morphology. It helps in detecting and managing conditions such as astigmatism, keratoconus, and post-surgical changes, facilitating appropriate treatment planning, including the fitting of contact lenses, refractive surgery evaluations, and disease progression monitoring.

The keratometry formula calculates the corneal power using the measurements from the anterior corneal surface. Thus, the corneal power in diopters is calculated using the following keratometry equation [36]:

(5) $$\Phi =\frac{337.5}{r}$$

where *r* represents the measured curvature radius in mm. This equation assumes a standard refractive index (*n* = 1.3375) for the cornea. Keratoconus causes a reduction in Placido ring symmetry and results in a steeper corneal curvature, leading to an increased diopter value. Thus, a significantly higher diopter reading in localized areas indicates the presence of keratoconus-related abnormalities.

### 2.3. Classification

For the classification step, we adopted the SVM algorithm by utilizing the sigmoid kernel function [37]. For the classification step, the images were divided into training and testing sets. The training set was later divided into training and validation subsets and the testing set was left out specifically for the testing procedure.

Thus, we adopt SVM to classify our samples ($x_{i}$) into healthy ($y_{i}$ = 1) and diseased ($y_{i}$ = −1). These two statuses are classified with the hyperplane ($f\left(x\right)$), which is defined as follows:

(6) $$f\left(x\right)=w^{T}\Phi \left(x\right)+b,$$

where b is bias, x is the input vector, Φ is the transform function, and w is the weight vector, which can be defined as

(7) $$w=\sum_{i=1}^{N}\alpha_{i}y_{i}{\Phi (x}_{i}),$$

where $\alpha_{i}$ ($\alpha_{i}>0)$ is the Lagrange multiplier to find the optimal hyperplane, and N is the training sample number. Consequently, the hyperplane decision can be written as follows:

(8) $$f\left(x\right)=\sum_{i=1}^{N}\alpha_{i}y_{i}{\Phi (x}_{i}).\Phi (x)+b,$$

The above equation can be written as

(9) $$f\left(x\right)=\sum_{i=1}^{N}\alpha_{i}y_{i}K\left(x_{i},x\right)+b,$$

where $K\left(.,.\right)$ is the kernel function. Here, we used the Gaussian kernel, which is defined as

(10) $$K\left(x_{i},x_{j}\right)=\exp\left(\frac{-{\left\|x-x_{i}\right\|}^{2}}{\sigma^{2}}\right),$$

where σ is the variance of the function.

The SVM optimal solution is acquired by maximizing the margin, which results in minimizing $||\left.w\right\|$. Therefore, we need to solve the following equation:

(11) $$\underset{\alpha_{i}}{\mathrm{argmin}}\sum_{i=1}^{n}\alpha_{i}-\frac{1}{2}\sum_{i,j=1}^{N}\alpha_{i}\alpha_{j}y_{i}y_{j}K\left(x_{i},x\right),$$

This is subject to 0 $\leq \alpha_{i}\leq$ C, and

(12) $$\sum_{i=1}^{N}\alpha_{i}y_{i}=0,$$

where C is a positive parameter that regulates the margin.

#### Cross-Validation

**Figure 7:**
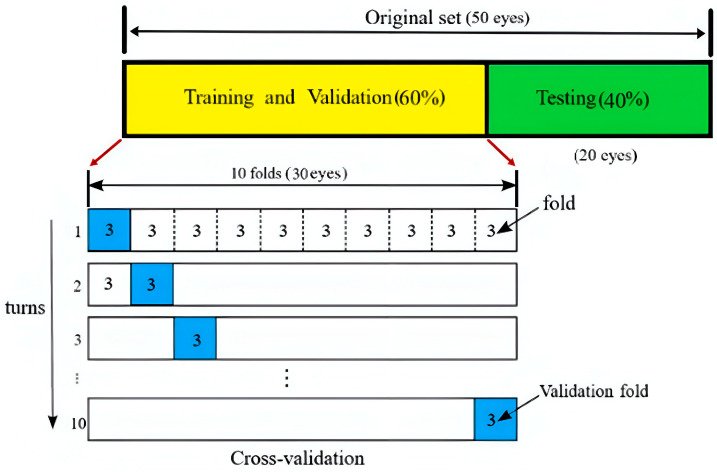
Ten-fold cross-validation method. In each turn, 1 fold is used as the validation fold and the remaining 9 folds are used for training. In total, 40% of the data are left out for testing the classifier.

To mitigate the risk of overfitting, we employed the k-fold cross-validation algorithm. To ensure a robust evaluation, 60% of the data were allocated for training and validation, while the remaining 40% were reserved for testing. A 10-fold cross-validation approach was employed to train and validate the classifier and minimize the risk of overfitting (refer to Figure 7). Each fold consisted of data from three subjects. In each iteration, one fold was designated for validation, while the remaining nine folds were used for training. This process was repeated over 10 iterations to ensure comprehensive model evaluation and validation.

## 3. Results

We experimented with multiple machine learning approaches, including Linear Support Vector Machine (SVM), Gaussian SVM, Convolutional Neural Networks (CNNs), and k-Nearest Neighbors (k-NN), to perform binary classification of the samples. The models achieved classification accuracies ranging from 90% to 97%, with the Gaussian SVM yielding the highest accuracy at 97%. While deep learning-based CNN models were explored, their computational intensity and high processing power requirements made them less suitable for practical implementation on a smartphone due to increased inference time, performance limitations, and excessive power consumption. Given the limited size of our dataset and the need for an efficient, lightweight, and fast model for mobile deployment, the SVM classification method provided better accuracy and more reliable detection results. The classification results, including the accuracy achieved by each machine learning method, are summarized in Table 2.

**Figure 8:**
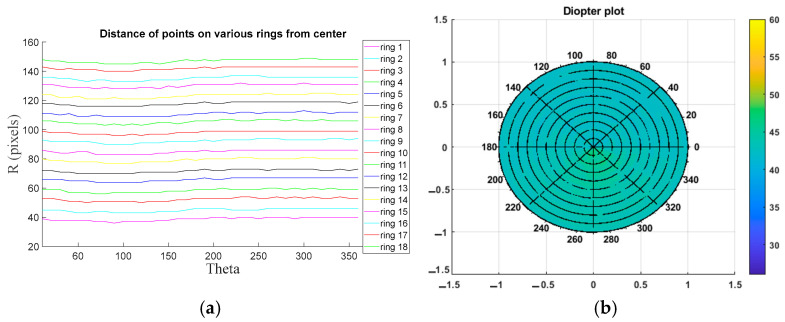
Results of Placido disc radius measurement and elevation map calculations on healthy eye: (**a**) distance of each of the 19 rings from the center of the Placido disc between 0 and 360 degrees and (**b**) diopter plot calculated using the radius map.

Figure 8 illustrates the results of disc radius measurements and elevation map calculations for a sample healthy eye based on the eye model. In Figure 8a, the measured distances for 18 rings from the center of the cornea, spanning 0 to 360 degrees, are presented. To enhance visualization, each ring is represented in a distinct color, with the measured distances displayed in pixel units. Figure 8b depicts the diopter plot of the corneal surface, calculated using the ring radii from Figure 8a. The diopter values are within the normal range, approximately +43D, indicating a healthy corneal surface.

**Figure 9:**
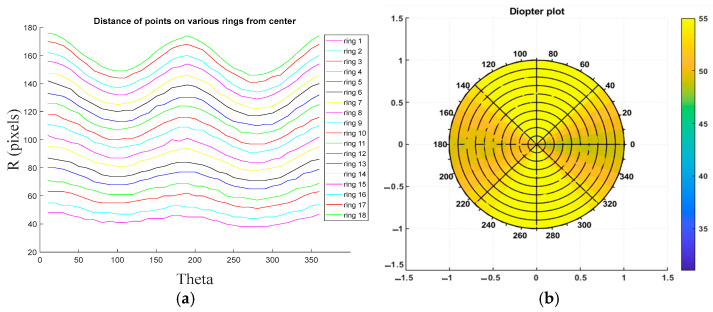
Results of the Placido disc radius measurement and elevation map calculations on a KC eye: (**a**) distance of each of the 19 rings from the center of the Placido disc between 0 and 360 degrees and (**b**) diopter plot calculated using the radius map.

Figure 9 presents the results of disc radius measurements and elevation map calculations for a sample diseased eye based on the eye model. The measurements of various ring radii, along with the corresponding curvature and diopter plots for the diseased eye, are illustrated. In Figure 9a, the measured ring radii are displayed, while Figure 9b shows the diopter plot of the corneal surface, calculated using the radii measurements from Figure 9a. The diopter plot reveals an inhomogeneous surface coloration (ranging from light yellow to dark yellow), indicative of an abnormal cone-shaped cornea, characteristic of a diseased condition.

**Figure 10:**
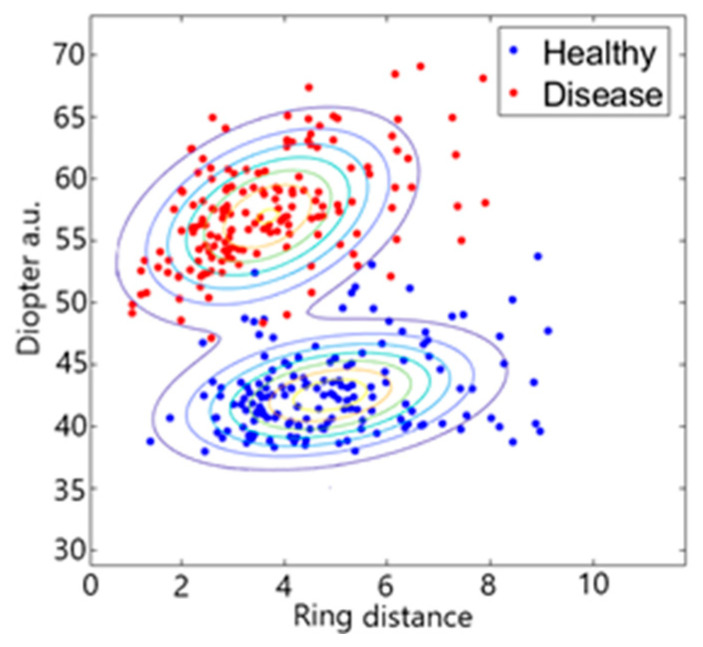
Scatter plot of fitted Gaussian contours: X-axis demonstrates the ring distance, and the Y-axis is the diopter of the cornea. The contour lines represent regions of equal probability density, illustrating the distribution of Healthy (blue) and Disease (red) data points.

**Figure 11:**
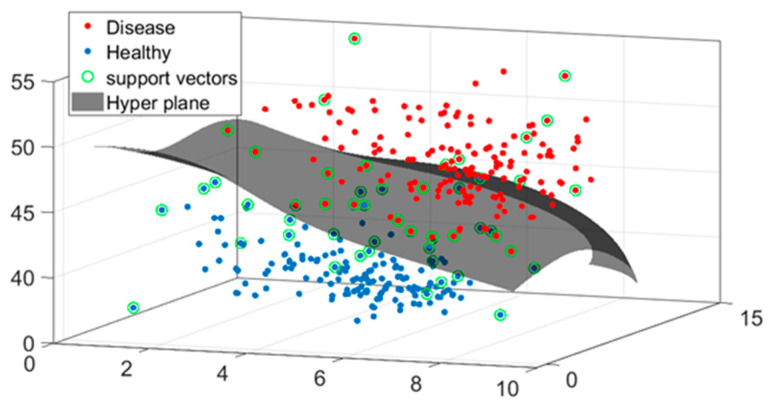
The results of the SVM classifier: Diseased eye features are shown in red and healthy eye features are shown in blue. The hyperplane is shown in gray, and the support vectors are shown in green.

After determining the radius of each Placido disc and calculating the corresponding diopter values, we employed a Support Vector Machine (SVM) classifier to differentiate between healthy and diseased corneas. Figure 10 presents the classification outcomes, visualized using a Gaussian scatter plot for two sample eyes. In the plot, features corresponding to diseased eyes are indicated in red, while features of healthy eyes are depicted in blue. The classification is based on the corneal diopter values and the distance from the center of the eye. The X-axis represents the ring number, and the Y-axis denotes the diopter values for the sampled healthy and diseased eyes. Figure 11 illustrates the results of the SVM classifier applied to two sample eyes (one healthy and one diseased), incorporating diopter values, the radius of curvature, and the ring distance from the center. In this figure, features of healthy eyes are marked in blue, and those of diseased eyes are shown in red. The support vectors are identified in green, and the hyperplane is represented in gray. As demonstrated in Figure 11, the keratoconic (KC) eye exhibits higher diopter values across various distances and radii compared to the healthy eye.

In keratoconus (KC) eyes, the diopter is elevated at the center of the cornea, resulting in a cone-like shape on the corneal surface. In contrast, healthy eyes exhibit a lower diopter at the center, with a gradual increase as one moves away from the center, creating a uniform dome-shaped corneal surface.

**Figure 12:**
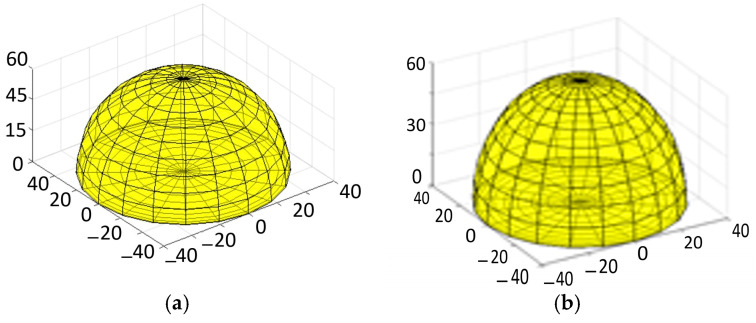
Comparison of healthy and diseased corneas: (**a**) a healthy cornea with a smooth dome shape, and (**b**) a diseased cornea with a conical shape that bulges outward.

Figure 12 illustrates the three-dimensional reconstruction of both healthy and diseased corneas. In Figure 12a, we see the 3D reconstruction of a healthy eye, which displays a uniform dome shape without any surface irregularities. In contrast, Figure 12b presents the 3D reconstruction of an eye affected by keratoconus (KC). This diseased eye exhibits a cone-shaped cornea that is slightly bulging outward. Consequently, as evidenced in Figure 11 and Figure 12, the eyes with KC have higher diopter values, leading to an elevation in the central part of the cornea and resulting in a pronounced cone shape compared to the normal cornea.

To evaluate the performance of our proposed method, we calculated the sensitivity, specificity, negative predictive value (NPV), positive predictive value (PPV), and accuracy using the following formulas:

(13) $$Sensitivity=\frac{TP}{TP+FN}\times 100\%$$

(14) $$Specificity=\frac{TN}{TN+FP}\times 100\%$$

(15) $$Accuracy=\frac{TP+TN}{TP+TN+FP+FN}\times 100\%$$

(16) $$NPV=\frac{TN}{TN+FN}\times 100\%$$

(17) $$PPV=\frac{TP}{TP+FP}\times 100\%$$

In this context, FP refers to false positives (healthy eyes mistakenly identified as diseased), TP denotes true positives (diseased eyes accurately diagnosed as diseased), FN indicates false negatives (diseased eyes incorrectly classified as healthy), and TN signifies true negatives (healthy eyes correctly identified as healthy).

Based on these definitions, our proposed method achieved sensitivity, specificity, and accuracy rates of 96.08%, 97.96%, and 97%, respectively. These findings demonstrate that the proposed method can effectively detect keratoconus (KC) with high accuracy using only a smartphone, eliminating the need for additional equipment.

## 4. Discussion

The findings presented in this study demonstrate the efficacy of a novel smartphone-based method for early keratoconus detection using Placido disc projections and machine learning classification. The proposed approach successfully addresses the key limitations of existing keratoconus diagnostic methods, such as reliance on expensive and bulky equipment, subjective analysis, and the need for skilled technicians. By leveraging the widespread availability and advanced imaging capabilities of modern smartphones, this method provides a cost-effective, portable, and user-friendly solution that could revolutionize keratoconus screening, particularly in remote or resource-constrained areas.

The integration of the CIE LAB color space for image preprocessing and feature extraction, combined with the Support Vector Machine (SVM) classifier, yielded highly accurate results. Achieving 96.08% sensitivity, 97.96% specificity, and 97% accuracy highlights the robustness of this approach for distinguishing between healthy and keratoconus-affected eyes. These metrics are on par with, or superior to, many existing diagnostic techniques that rely on more complex and less accessible technologies.

This study also underscores the importance of preprocessing steps such as image dehazing and contrast enhancement in improving the clarity and reliability of diagnostic images. The use of an SVM classifier with a Gaussian kernel function, optimized through 10-fold cross-validation, demonstrates the potential of machine learning algorithms to reduce bias and enhance diagnostic precision. The classification results, particularly the separation between healthy and diseased corneal features, support the feasibility of this method for early detection of keratoconus.

To provide a comprehensive evaluation, we compare our proposed method with state-of-the-art keratoconus detection techniques, including traditional corneal topography, Scheimpflug imaging, and existing smartphone-based solutions. Conventional corneal topography techniques, such as Placido-based systems, rely on large and expensive clinical devices, making them inaccessible in non-specialized settings. Similarly, Scheimpflug imaging provides highly accurate 3D mapping of the corneal structure but requires trained personnel and costly equipment, limiting its deployment outside specialized clinics. In contrast, our method offers a cost-effective and portable alternative by utilizing a smartphone’s built-in display and camera, significantly improving accessibility.

Existing smartphone-based approaches often require external attachments, such as 3D-printed Placido discs or slit-lamp adapters, which increase complexity and cost. Additionally, some methods rely on single-image processing techniques that lack robustness due to variations in illumination and positioning. Our solution overcomes these limitations by providing a fully standalone system that eliminates the need for external attachments while integrating adaptive contrast adjustments and machine learning-based classification, ensuring higher accuracy and usability.

The proposed method presents several advantages, including the elimination of additional hardware dependencies, reduced cost by leveraging widely available smartphone technology, and the use of adaptive contrast mechanisms to enhance image clarity under varying lighting conditions. With a high accuracy rate of 97%, it outperforms existing smartphone-based solutions and can be easily deployed in resource-limited settings, increasing accessibility for early keratoconus detection.

Despite its promising results, this study has limitations that warrant further investigation. The dataset, while diverse in terms of disease stages, is relatively small, which may affect the generalizability of the findings. Expanding the dataset to include a larger number of real patient images, as opposed to emulated eye models, would strengthen the clinical applicability of this method. Additionally, while the proposed approach eliminates the need for external devices, future studies should assess its performance across a range of smartphone models with varying camera specifications to ensure consistency and reliability. Another potential area for improvement involves automating the placement and alignment of the Placido discs to further simplify the process for non-expert users. Future iterations of the method could incorporate advanced computational techniques or AI-driven automation to enhance user experience and reduce variability.

The proposed smartphone-based keratoconus detection method represents a significant step forward in democratizing access to ophthalmic diagnostics. By combining innovative image processing techniques with machine learning, this approach has the potential to improve early detection rates, facilitate timely interventions, and ultimately reduce the global burden of keratoconus-related blindness. Further research and development, including large-scale clinical validation, will be essential to transition this promising technology from the laboratory to widespread clinical and public health applications.

## 5. Conclusions

In this paper, we present a novel method for detecting keratoconus (KC) that utilizes only a single smartphone, without the need for any additional gadgets or attachments. We evaluated our proposed technique across four different stages of KC, simulated using an eye model. Our method involves capturing images with various smartphone cameras from a total of 100 eye models, consisting of 50 with KC and 50 healthy ones. The images were obtained from eye models that replicate different stages of KC disease as well as healthy eyes. Data acquisition was performed using the smartphone’s front camera, while the smartphone screen was employed to project Placido discs onto the corneal surface. During the preprocessing stage, we applied dehazing and contrast enhancement algorithms. Using the CIE LAB color space, we extracted the projected Placido discs and subsequently calculated their radii and diopters. In the final step, a Support Vector Machine (SVM) classifier was used to differentiate KC eyes from healthy ones. Our results demonstrate that the proposed method achieves a KC detection accuracy of 97%, along with a specificity of 97.96% and sensitivity of 96.08%.

## Figures and Tables

**Table 1:** Extracted values of the Placido discs projected on the cornea.

Color Channel	L	A	B
Mean ± SD	50.449 ± 3.8	73.090 ± 4.6	51.361

**Table 2:** Comparison between the performance of four different classifiers.

Classifier	Linear SVM	Gaussian SVM	k-NN	CNN
Accuracy	93%	97%	90%	92%

## Data Availability

The raw data supporting the conclusions of this article will be made available by the authors on request.
